# Laminarin-induced apoptosis in human colon cancer LoVo cells

**DOI:** 10.3892/ol.2014.1952

**Published:** 2014-03-07

**Authors:** CHEN-FENG JI, YU-BIN JI

**Affiliations:** 1 Engineering Research Center of Natural Anticancer Drugs, Ministry of Education, Harbin, Heilongjiang 150076, P.R. China; 2 Center of Research on Life Science and Environmental Science, Harbin University of Commerce, Harbin, Heilongjiang 150076, P.R. China

**Keywords:** human colon cancer, apoptosis, mechanism, laminarin

## Abstract

A number of scientific studies have revealed that laminarin has antitumor effects. Therefore, the aim of the present study was to investigate the apoptosis of LoVo cells and the underlying mechanisms induced by laminarin. LoVo cells were treated with various concentrations of laminarin and fluorescence-inverted microscopy was used to observe the morphology of LoVo cells treated with laminarin. In addition, western blotting was performed to analyze the expression levels of death receptor (DR)4, DR5, TNF-related apoptosis-inducing ligand (TRAIL), Fas-associated protein with death domain (FADD), caspase-8, caspase-3, Bid and tBid. Flow cytometry was conducted to analyze the expressions of Bcl-2 and Bax, and spectrophotometry was performed to quantify the activity of caspases-8, -3, -6 and -7. Following the treatment of LoVo cells with laminarin for 24 h, the expression levels of DR4, DR5, TRAIL, FADD, Bid, tBid and Bax were observed to be upregulated, whereas the expression levels of pro-caspase-8, pro-caspase-3 and Bcl-2 were downregulated. In addition, the activities of casapse-8, -3, -6 and -7 were observed to increase, which was a significant difference when compared with those of the control group. Therefore, laminarin is considered to induce the apoptosis of LoVo cells, which may occur via a DR pathway, suggesting that laminarin may be a potent agent for cancer treatment.

## Introduction

Colon cancer is a common malignant tumor of the digestive tract and one of the four most common types of malignant tumors worldwide, and therefore presents a significant global health problem. Despite recent advances in the chemotherapy treatment for colon cancer, the outcomes of anticancer therapy remain unsatisfactory. Thus, further improvement of therapies for colon cancer is required. Numerous pharmacological studies have shown that polysaccharides from certain traditional Chinese medicines exhibit antitumor effects with fewer side effects.

Laminarin is an active component which is extracted and isolated from the dry thallus of *Laminaria japonica* Aresch of the Laminariaceae family, or *Ecklonia kurome* Okam of the Araliaceae family (1). Laminarin consists of β-(1–3)-glucan with β-(1–6)-linkages. The antitumor effect of laminarin has been previously reported (2–5), and Park *et al* demonstrated that laminarin inhibits HT-29 cell growth by decreasing cell proliferation and inducing apoptosis via the death receptor (DR) and insulin-like growth factor I receptor pathways (6).

Our previous study revealed that laminarin increases the intracellular levels of reactive oxygen species and Ca^2+^, decreases intracellular pH levels and induces apoptosis in LoVo cells. In addition, laminarin was observed to open mitochondrial permeability transition pores (MPTPs), activating the death switch and subsequently decreasing the mitochondrial membrane potential, thus inducing apoptosis through an irreversible mitochondrial pathway. Furthermore, laminarin may alter the expression of apoptosis-related proteins, such as cytochrome *c* (cyt *c*), caspase-9 and caspase-3, in LoVo cells and induce apoptosis. Therefore, it may be hypothesized that laminarin induces apoptosis in human colon cancer LoVo cells through a mitochondrial pathway (7).

Predominantly, apoptosis may be initiated in two ways, via an intrinsic (mitochondrial-mediated) or extrinsic (DR-mediated) pathway (8–10). Each pathway exhibits various interactions, which ultimately lead to apoptosis. Based on the observations of our previous study, the present study further investigated whether laminarin may induce apoptosis in LoVo cells via an alternative apoptosis pathway, the DR pathway, which has not previously been reported. In addition, this study may increase the development and application of laminarin for colon cancer treatment.

## Materials and methods

### Main reagents

The following reagents were used: Laminarin and paraformaldehyde (Sigma-Aldrich, St. Louis, MO, USA); hydroxycamptothecin (HCPT, Harbin Shengtai Pharmaceutical Co., Ltd., Harbin, China); Dulbecco’s modified Eagle’s medium (DMEM)/F12 culture medium (Thermo Fisher Scientific Inc., Waltham, MA, USA); fetal bovine serum (FBS; Hangzhou Sijiqing Biological Engineering Materials Co., Ltd., Hangzhou, China); pancreatin (Gibco-BRL, Rockville, MD, USA); Hoechst 33258 (Sigma-Aldrich); rabbit anti-human β-actin polyclonal antibody, DR4, DR5, TNF-related apoptosis-inducing ligand (TRAIL), Fas-associated protein with death domain (FADD) and Bid (Biosynthesis Biotechnology Co., Ltd., Beijing, China); mouse anti-human Bcl-2, rabbit anti-human Bax and fluorescein isothiocyanate (FITC)-goat anti-mouse polyclonal antibodies (Boster Biological Technology Co., Ltd., Wuhan, China); mouse anti-human caspase-8 and -3, alkaline phosphatase goat anti-rabbit polyclonal antibodies, as well as caspase-8, -3, -6 and -7 activity assay kits, sodium dodecyl sulphate sample buffer, polyacrylamide gel, nitrocellulose membranes, 5-bromo-4-chloro-3-indolyl-phosphate (BCIP)/nitroblue tetrazolium (NBT) alkaline phosphatase color development kit, Triton X-100, bovine serum albumin and phosphate-buffered saline (PBS; Beyotime Institute of Biotechnology, Haimen, China); detergent-compatible protein assay kit (Bio-Rad, Hercules, CA, USA).

### Main apparatus

The CKX41 fluorescence inverted microscope was purchased from Olympus (Tokyo, Japan), and the Mini-Protean Tetra and Mini Trans-Blot electrophoresis systems, Gel Doc XR imaging system and Model 680 microplate reader were purchased from Bio-Rad. The EPICS XL flow cytometer was obtained from Beckman Coulter (Miami, FL, USA) and the CO-150 CO_2_ incubator was purchased from New Brunswick Scientific (Edison, NJ, USA). The UV1000 UV-VIS spectrophotometer was purchased from Techcomp Limited (Shanghai, China).

### Cell culture

Human colon cancer LoVo cell lines were provided by the Center of Research and Development on Life Sciences and Environmental Sciences of Harbin University of Commerce (Harbin, China). The LoVo cells were cultured in DMEM/F12 medium containing 10% heat-inactivated FBS at 37°C in a humidified atmosphere of 5% CO_2_.

### Effect of laminarin on LoVo cell morphology

In total, 5×10^4^ cells were seeded in six-well plates, cultured for 24 h and treated with various concentrations of laminarin for 72 h. Cells were then harvested by trypsinization, washed twice with cold PBS and fixed in 4% paraformaldehyde for 30 min at 4°C. The fixing solution was then discarded and the cells were washed twice with PBS. Next, the cells were stained with Hoechst 33258 for 20 min. The stain was discarded and cells were washed twice with PBS prior to observation under a fluorescence microscope.

### Effect of laminarin on the expression of DR4, DR5, TRAIL, FADD, caspase-8, caspase-3, Bid and tBid in LoVo cells

In total, 5×10^4^ cells/ml (2 ml) were seeded in six-well plates and cultured for 24 h, followed by treatment with various concentrations of laminarin for 48 h. The cytoplasm extracts were prepared with 150 μl cell lysis buffer on ice for 30 min. The solution was then centrifuged at 10,000 × g for 10 min and the supernatant was collected. The protein concentration was quantified using the detergent-compatible protein assay kit. Next, the proteins were mixed with 2X sodium dodecyl sulphate sample buffer and a total of 40 μg of protein was separated in 10% (w/v) polyacrylamide gel and blotted onto nitrocellulose membranes. The blots were blocked for 2 h and incubated with the primary antibody for 12 h. Subsequently, the membranes were washed in buffer and incubated with the secondary antibody in blocking buffer. Ponceau staining was performed to ensure equal loading and the bands were detected by BCIP/NBT alkaline phosphatase color development kit. The bands were then visualized and quantified using the Gel Doc XR imaging system.

### Effect of laminarin on the activity of caspase-8, -3, -6 and -7 in LoVo cells

A total of 5×10^4^ cells were seeded in 24-well plates, cultured for 24 h and treated with various concentrations of laminarin for 24 h. The cells were then digested with pancreatin and rinsed twice with PBS. Caspase activity was determined by colorimetric assay using the previously mentioned kits, according to the manufacturer’s instructions. The optical density of the reaction mixture was quantified using a spectrophotometer at a wavelength of 405 nm.

### Effect of laminarin on the expression of Bcl-2 and Bax in LoVo cells

A total of 5×10^4^ cells were seeded in six-well plates, cultured for 24 h and treated with various concentrations of laminarin for 24 h. Cells were then digested with pancreatin and rinsed twice with PBS. Next, 2 ml paraformaldehyde (40 g/l) was added to fix cells for 40 min. The fixing solution was then removed and cells were rinsed twice with PBS. In total, 1 ml Triton X-100 (0.1%) was added for 15 min to punch holes in the cell membranes. The Triton X-100 was then removed and the cells were rinsed with PBS twice. Next, 1 ml bovine serum albumin (1%) was added to seal cells for 1 h. The sealing liquid was then removed and mouse anti-human Bcl-2 and Bax antibodies were added to cells and incubated for 1 h at 37°C. The supernatant fluid was then removed and cells were rinsed with PBS. Next, FITC anti-mouse antibody was added and cells were incubated for 30 min at room temperature. Finally, the supernatant fluid was discarded and 500 μl PBS was added prior to analysis of the cells by flow cytometry (FCM).

### Statistical analysis

Data are presented as the mean ± standard deviation. Statistical analyses were performed using the analysis of variance test to compare the different groups. P<0.05 was considered to indicate a statistically significant difference.

## Results

### Effect of laminarin on LoVo cell morphology

**Figure 1:**
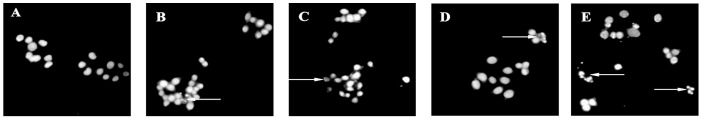
Effect of laminarin on LoVo cell morphology. LoVo cells were treated with (A) Dulbecco’s modified Eagle’s medium/F12 culture medium as a control and (B) hydroxycamptothecin (5 μg/ml) as a negative control, as well as (C) low (400 μg/ml), (D) middle (800 μg/ml) and (E) high (1600 μg/ml) concentrations of laminarin. Arrows indicate apoptotic bodies.

Inverted fluorescence microscopy revealed that cells in the control group grew normally, with cells adhering to the bottom of the plate. The shapes of the endochylema were fusiform, polygonal and irregular with round cell nuclei. Following treatment with various concentrations of laminarin for 72 h, a high proportion of cells demonstrated apoptosis-like changes, including detachment and cytoplasmic condensation, which lead to cellular swelling, rounding, disappearance of the microvilli and cytoplasmic condensing. Treatment with HCPT as a positive control drug for 72 h caused the cytoplasm to condense and apoptotic bodies appeared in the LoVo cells. Consequently, the number of apoptotic bodies was observed to increase (Fig. 1).

### Effect of laminarin on the expression of DR4, DR5, TRAIL, FADD, caspase-8 and caspase-3 in LoVo cells

**Figure 2:**
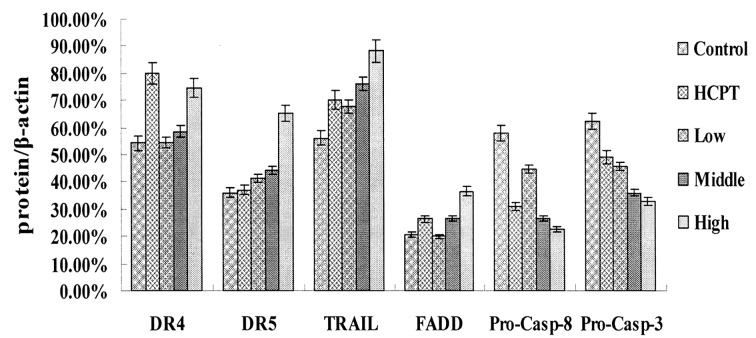
Effect of laminarin on apoptosis-related protein expression in LoVo cells. The LoVo cells were treated with low (400 μg/ml), middle (800 μg/ml) and high (1,600 μg/ml) concentrations of lamarin, as well as Dulbecco’s modified Eagle’s medium/F12 culture medium as a control and HCPT as a negative control. DR, death receptor; TRAIL, TNF-related apoptosis-inducing ligand; FADD, FAS-associated protein with death domain; Casp, caspase; HCPT, hydroxycamptothecin.

**Figure 3:**
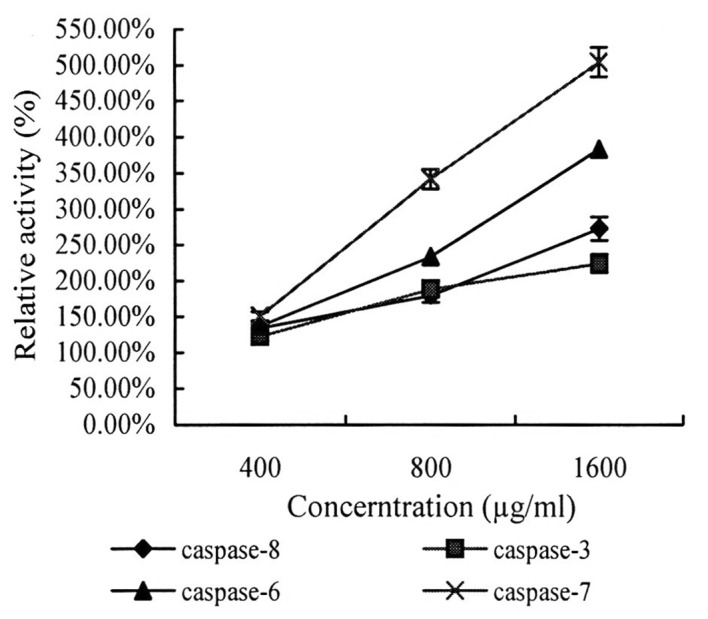
Effects of laminarin on caspase activity in LoVo cells.

The results of the western blotting demonstrated that following treatment with laminarin for 24 h, the expression levels of DR4, DR5, TRAIL and FADD increased, whereas the expression levels of procaspase-8 and -3 decreased. This effect was dose-dependent and the expression levels were significantly different compared with those of the control group (Figs. 2 and 3).

Treatment with HCPT as a positive control drug for 24 h, caused the expression levels of DR4, TRAIL and FADD in the LoVo cells to increase markedly and the expression levels of procaspase-8 and-3 to decrease markedly compared with the control group (Fig. 2).

### Effect of laminarin on the activity of caspase-8, -3, -6 and -7 in LoVo cells

The results showed that following treatment with laminarin for 24 h, the concentration of pentose nucleic acid in LoVo cells had increased and was significantly different compared with that of the control group and, subsequently, caspase activity increased. Following treatment with laminarin at concentrations of 400, 800 and 1,600 μl/ml, caspase activity increased by 34.32, 80.09 and 172.81% for caspase-8; 2.84, 15.77 and 22.92% for caspase-3; 37.04, 153.77 and 283.49% for caspase-6; and 50.44, 242.44 and 434.44% for caspase-7, respectively. Thus, caspase activity was observed to increase in a dose-dependent manner.

### Effect of laminarin on the expression of Bid, tBid, Bcl-2 and Bax in LoVo cells

**Figure 4:**
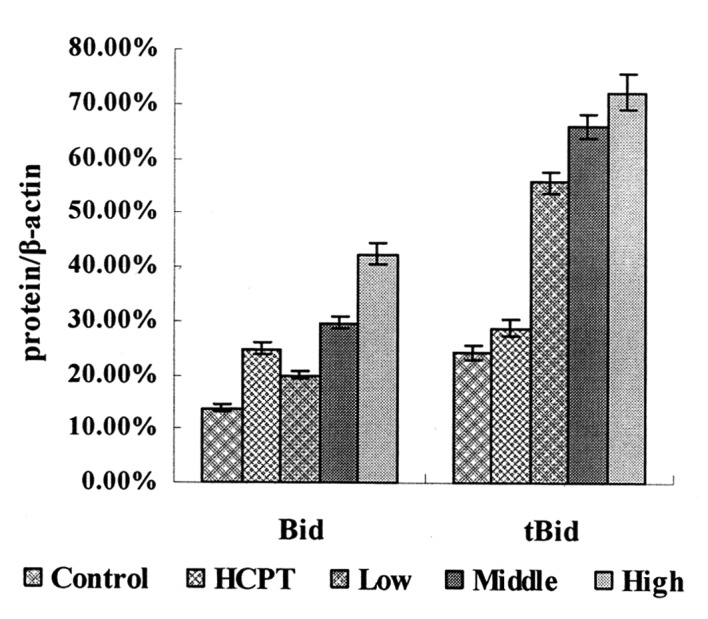
Effect of laminarin on Bid and tBid expression in LoVo cells. The LoVo cells were treated with low (400 μg/ml), middle (800 μg/ml) and high (1,600 μg/ml) concentrations of lamarin, as well as Dulbecco’s modified Eagle’s medium/F12 culture medium as a control and HCPT as a negative control. HCPT, hydroxycamptothecin.

Western blotting showed that following laminarin treatment for 24 h, the expression levels of Bid and tBid in LoVo cells increased in a concentration-dependent manner and were significantly different compared with those in the control group (Fig. 4).

Treatment with HCPT as a positive control drug for 24 h, led to an increase in the expression levels of Bid and tBid in the LoVo cells, additionally the expression levels of Bid were markedly different compared with the control group (Fig. 4).

The FCM results demonstrated that following treatment with laminarin for 24 h, Bcl-2 expression levels had decreased, whereas Bax expression levels had increased, in a concentration-dependent manner. The expression levels of Bcl-2 and Bax in the laminarin treatment group were significantly different from those in the control group (Table I).

Treatment with HCPT as positive control drug for 24 h led to a marked increase in the expression level of Bax in LoVo cells and the expression level of Bcl-2 decreased markedly compared with the control group (Table I).

## Discussion

Apoptosis is a rigorous, active and orderly process of cell death that is regulated by numerous genes to maintain the stability of the intracellular environment (11). The DR pathway is one of the three major apoptosis pathways. Significant DRs include Fas, tumor necrosis factor (TNF) receptor 1 and DR3–6. The DRs, when combined with their respective ligands, mediate cell apoptosis (12).

TRAIL is a member of the TNF superfamily. The signal transmission pathway for apoptosis, induced by TRAIL and its receptors, is characterized by selective promotion of tumor cell apoptosis (13,14). The mechanism by which TRAIL and its receptors induce apoptosis is as follows: DR4 and DR5 are functional receptors that contain death domains (DDs), which, when combined with TRAIL, transmit signals for apoptosis into the cell. The DD on the C-terminus of FADD then interacts with that on DR4/DR5. The N-terminus of FADD also contains a DD, which mediates the signal transmission for apoptosis. When the DD is combined with the death effectors domain on procaspase-8, the TRAIL-DR4/DR5-FADD-procaspase-8/death-inducing signaling complex (DISC) is formed. The procaspase-8 in DISC cleaves itself to produce active caspase-8, which activates two pathways for apoptosis signaling. In the first pathway, caspase-8 directly activates caspase-3, -6 and -7, which induces apoptosis via the DR pathway (15,16). In the second pathway, caspase-8 connects to the mitochondrion via the activation of Bid, which subsequently induces apoptosis through the mitochondrion (17,18).

HCPT is an agent with an unique spectrum of anti-tumor activity, it may significantly inhibit cell proliferation and induce apoptosis in colon cancer through both intrinsic and extrinsic pathways (19). In this study, it was used as a positive drug and it was demonstrated that HCPT was capable of inhibiting LoVo cell proliferation and also inducing apoptosis through extrinsic (DR-mediated) apoptotic pathways, which may upregulate the expression of DR4, TRAIL and FADD and downregulate the expression of procaspase-8 and-3, resulting in the activation of the caspase enzyme and apoptosis.

The results of the western blotting showed that laminarin increases the expression of DR4, DR5, TRAIL and FADD in LoVo cells and decreases the expression of procaspase-8 and -3 in a dose-dependent manner and the effects are the same with HCPT. This suggests that laminarin promotes DISC formation in LoVo cells, which induces apoptosis via a death receptor pathway that is mediated by TRAIL/DR4/DR5.

Caspase-8 activates two apoptotic pathways, and the results of the current study confirm that laminarin increases caspase-8 activity and induces LoVo cell apoptosis via the DR pathway; caspase-8 activates caspase-3, -6 and -7, thus inducing apoptosis. However, caspase-8 is also associated with mitochondria via the activation of Bid, which subsequently induces apoptosis through the mitochondria.

Bid is an apoptotic protein of the Bcl-2 family that has a BH3 structural domain and is important in the mitochondrial and DR pathways of cell apoptosis, and is usually termed the ‘hub’ or the ‘crosstalk regulation’ (20,21). Under normal physiological conditions, Bid is located in the cytoplasm in an inactive state; however, when the cell surface DRs are activated, the Bid proteins are cleaved into 15-kDa functional tBid fragments, which are repositioned on the mitochondrial membrane where they cooperate with Bax proteins. This cooperation promotes the fusion of Bax and the mitochondria, which results in changes in the configuration of Bax proteins. These changes increase damage to the mitochondria, which results in the formation of membrane pores and allows large amounts of cyt *c* to be released from the mitochondria. Subsequently, caspase-9 is activated and leads to the induction of apoptosis in the cells (22,23). In addition, tBid binds to Bcl-2 and inhibits the anti-apoptosis effect (24,25).

Bcl-2 family members are important in the regulation and control of the apoptosis pathway, and are divided into anti-apoptotic and pro-apoptotic proteins. The regulation of apoptosis involves targeting of the mitochondria by anti- and pro-apoptotic Bcl-2 proteins. Bcl-2 inhibits MPTP opening and prevents apoptosis. In addition, Bcl-2 can directly or indirectly prevent cyt *c* release and form the Bcl-2-Apaf1-caspase-9 complex, resulting in an anti-apoptosis effect. However, Bax can promote MPTP opening and the subsequent cyt *c* release, which activates the caspase cascade reaction, eventually resulting in apoptosis.

The results of the current study demonstrated that laminarin increases the protein expression of Bid, tBid and Bax (as well as its apoptosis-promoting effects). In addition, laminarin was identified to reduce the protein expression of Bcl-2 and its anti-apoptosis effect in LoVo cells, and may transmit signals for apoptosis to the mitochondria through the Bid hub. Our previous study revealed that laminarin induces the opening of MPTPs in LoVo cells, which lowers the mitochondrial membrane potential and consequently increases the expression of cytoplasmic cyt *c* (7). This significantly increases the expression and activity of caspase-9 and -3. The results of the present study closely correlate with those from our previous study, suggesting that laminarin may induce apoptosis in LoVo cells via two pathways: The DR and mitochondrial pathways.

In conclusion, laminarin upregulates DR4, DR5, TRAIL and FADD expression levels in LoVo cells, downregulates procaspase-8 and -3 expression levels and increases the activity of caspase-8, -3, -6 and -7. Therefore, laminarin may induce apoptosis in human colon cancer LoVo cells via the TRAIL/DR pathway. Laminarin also alters Bid, tBid, Bcl-2 and Bax expression levels in LoVo cells, which have close interactions with the mitochondrial pathway. Thus, the present study indicates that laminarin induces apoptosis in LoVo cells via the mitochondrial and DR pathways, suggesting that laminarin is a potent agent for cancer treatment. In addition, laminarin may be used for the therapy and prevention of certain types of digestive tract cancers and therefore, drug preparations must be determined for future clinical application.

## Figures and Tables

**Table I:** Effect of laminarin on Bcl-2 and Bax expression in LoVo cells.

		Mean fluorescence intensity (mean ± SD)	
		
Groups	Concentration μg/ml	Bcl-2	Bax
Control	0	25.4±0.84	14.4±0.73
HCPT	5	24.0±0.65^a^	19.4±0.66^b^
	400	24.8±0.89	14.8±0.58
Laminarin	800	23.3±0.93^b^	15.6±0.98^a^
	1600	21.6±0.56^b^	17.0±0.83^b^

a P<0.05 and

b P<0.01 vs. control group.

HCPT, hydroxycamptothecin; SD, standard deviation.

## References

[1] Yuan XL, Lv JZ, Xiao J, Ma Q (2010). Research progress of Laminarin. An Hui Nang Ye Ke Xue Bian Ji Bu.

[2] Ji CF, JI YB, Meng DY (2013). Sulfated modification and anti-tumor activity of laminarin. Exp Ther Med.

[3] Fuentes AL, Millis L, Sigola LB (2011). Laminarin, a soluble beta-glucan, inhibits macrophage phagocytosis of zymosan but has no effect on lipopolysaccharide mediated augmentation of phagocytosis. Int Immunopharmacol.

[4] Lee JY, Kim YJ, Kim HJ (2012). Immunostimulatory effect of laminarin on RAW 264.7 mouse macrophages. Molecules.

[5] Kim EJ, Lee YJ, Shin HK, Yoon JH (2006). A study on the mechanism by which the aqueous extract of *Inonotus obliquus* induces apoptosis and inhibits proliferation in HT-29 human colon cancer cells. J Korean Soc Food Sci Nutr.

[6] Park HK, Kim IH, Kim J, Nam JJ (2012). Induction of apoptosis by laminarin, regulating the insulin-like growth factor-IR signaling pathways in HT-29 human colon cells. Int J Mol Med.

[7] Ji YB, Ji CF, Zhang H (2012). Laminarin induces apoptosis of human colon cancer LOVO cells through a mitochondrial pathway. Molecules.

[8] Brenner D, Mak TW (2009). Mitochondrial cell death effectors. Curr Opin Cell Biol.

[9] Jeong SY, Seol DW (2008). The role of mitochondria in apoptosis. BMB Rep.

[10] Mellier G, Huang S, Shenoy K, Perraiz S (2010). TRAILing death in cancer. Mol Aspects Med.

[11] Krysko DV, Vanden Berghe T, D’Herde K, Vandenabeele P (2008). Apoptosis and necrosis: detection, discrimination and phagocytosis. Methods.

[12] Kleinberg L, Davidson B (2009). Cell survival and apoptosis-related molecules in cancer cells in effusions: a comprehensive review. Diagn Cytopathol.

[13] Hori T, Kondo T, Kanamori M (2010). Ionizing radiation enhances tumor necrosis factor-related apoptosis-inducing ligand (TRAIL)-induced apoptosis through up-regulations of death receptor 4 (DR4) and death receptor 5 (DR5) in human osteosarcoma cells. J Orthop Res.

[14] Szliszka E, Mazur B, Zydowicz G (2009). TRAIL-induced apoptosis and expression of death receptor TRAIL-R1 and TRAIL-R2 in bladder cancer cells. Folia Histochem Cytobiol.

[15] Pennarun B, Meijer A, de Vries EG (2010). Playing the DISC: turning on TRAIL death receptor-mediated apoptosis in cancer. Biochim Biophys Acta.

[16] Mazurek N, Byrd JC, Sun YJ, Bresalier R (2009). W1930 nuclear galectin-3 confers tumor necrosis factor-related apoptosis-inducing ligand (TRAIL) resistance to colon cancer cells by inhibiting Caspase-8 activation. Gastroenterology.

[17] Naumann I, Kappler R, von Schweinitz D (2011). Bortezomib primes neuroblastoma cells for TRAIL-induced apoptosis by linking the death receptor to the mitochondrial pathway. Clin Cancer Res.

[18] Kantari C, Walczak H (2011). Caspase-8 and bid: caught in the act between death receptors and mitochondria. Biochim Biophys Acta.

[19] Fei BJ, Chi AL, Weng Y (2013). Hydroxycamptothecin induces apoptosis and inhibits tumor growth in colon cancer by the downregulation of survivin and XIAP expression. World J Surg Oncol.

[20] Ghiotto F, Fais F, Bruno S (2010). BH3-only proteins: the death-puppeteer’s wires. Cytometry A.

[21] Repnik U, Turk B (2010). Lysosomal-mitochondrial cross-talk during cell death. Mitochondrion.

[22] Song G, Chen GG, Hu T, Lai PB (2010). Bid stands at the crossroad of stress-response pathways. Curr Cancer Drug Targets.

[23] Golbano JM, Lóppez-Aparicio P, Recio MN, Pérez-Albarsanz MA (2008). Finasteride induces apoptosis via Bcl-2, Bcl-xL, Bax and caspase-3 proteins in LNCaP human prostate cancer cell line. Int J Oncol.

[24] Shore GC, Nguyen M (2008). Bcl-2 proteins and apoptosis: choose your partner. Cell.

[25] Zhang ZF, Guo Y, Zhang JB, Wei XH (2011). Induction of apoptosis by chelerythrine chloride through mitochondrial pathway and Bcl-2 family proteins in human hepatoma SMMC-7721 cell. Arch Pharm Res.

